# Localized Temperature Monitoring in Mouse Brain during Light Delivery via a Non‐Planar Tapered Fiber‐Integrated µRTD Sensor

**DOI:** 10.1002/adma.202519655

**Published:** 2026-04-02

**Authors:** Antonio Balena, Marco Bianco, Barbara Spagnolo, Muhammad Fayyaz Kashif, Alberto Bramati, Massimo De Vittorio, Ferruccio Pisanello

**Affiliations:** ^1^ Istituto Italiano di Tecnologia–Center for Biomolecular Nanotechnologies Arnesano (LE) Italy; ^2^ Laboratoire Kastler Brossel Sorbonne University CNRS ENS‐PSL University Collège de France Paris France; ^3^ Università Degli Studi di Napoli Federico II Dipartimento di Ingegneria Elettrica e delle Tecnologie dell'Informazione Napoli Italy; ^4^ Università del Salento–Dipartimento di Ingegneria dell'Innovazione Lecce (LE) Italy

**Keywords:** multifunctional neural interfaces, optogenetics, tapered optical fibers, temperature monitoring, two‐photon polymerization

## Abstract

Monitoring local brain temperature with high spatial precision is essential to understanding neurophysiological processes and managing the side effects of optical neuromodulation techniques. We present a novel multifunctional neural interface integrating a microscale resistance temperature detector (µRTD) onto the curved surface of a tapered optical fiber (TF), enabling co‐localized light delivery and thermal sensing with minimal footprint. The µRTD, patterned via an unconventional two‐photon polymerization (TPP)‐based process on the fiber surface, exhibits thermal sensitivity <0.1°C and low self‐heating under physiologically‐safe bias conditions. We demonstrate the system's capacity to resolve subtle temperature changes induced by optogenetic stimulation/inhibition protocols (the latter requiring illumination periods of hundreds of milliseconds up to several seconds), revealing significant thermal accumulation only under long, high‐intensity illumination. This integration resolves the spatial mismatch of multimodal probes and reduces implant cross‐section compared to coaxial or side‐by‐side configurations. Furthermore, the TPP approach is modular, allowing integration with additional functionalities (i.e., electrophysiological recording or thermoplasmonics). By uniting photonic and thermal readout into a minimally invasive probe, our technology offers a powerful tool for studying thermally mediated neural processes, enhancing the safety and interpretability of optical neurotechnologies. Its integration potential positions this platform as a complementary technology for next‐generation multifunctional neural interfaces.

## Graphical abstract

We report a multifunctional tapered optical fiber integrating a conformal micro‐resistance temperature detector (µRTD) for local, real‐time thermometry during optical stimulation. The platform combines light‐delivery and temperature sensing within a minimally invasive footprint, enabling detection of sub‐degree cortical heating under representative protocols. Calibration, reproducibility, and artifact mitigation are demonstrated, providing a practical route toward integrated thermal monitoring in neural interfaces.

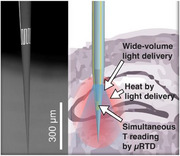

## 1 Introduction

Monitoring temperature variations in the mammalian brain aids in understanding various aspects of neural activity and functioning [1], as these variations can be directly linked to changes in neural activity or both endogenous and exogenous variations [2, 3, 4]. In physiological conditions, temperature increase has been linked to disruption of the blood–brain barrier, affecting brain homeostasis, brain hydration and ionic balance [5]. In the case of neurological diseases or traumatic brain injury [6, 7], changes in brain temperature have also been observed in specific regions. In addition, thermal approaches to treat brain cancer are playing a growing role in neuro‐oncology [8], and their further development would greatly benefit from the ability to measure local temperature gradients. Indeed, temperature increases should be confined to very small volumes affected by the lesions, while the temperature of the surrounding and healthy tissue remains unchanged [9]. Although noninvasive methods successfully provide real‐time temperature detection in this framework [10, 11], they suffer from reduced spatial resolution, thus limiting their use in measurements in specific and deep brain regions, where high spatial resolution is demanded, especially in small rodents. Complementarily, implantable probes based on thermocouples, thermistors, semiconductor‐based sensors, and Resistance Temperature Detectors (RTD) have proven their reliability in intracranial localized temperature measurements. However, in their current implementations – typically cables or substrate‐patterned – they face challenges related to implant size, integration with stimulation and readout modalities, as well as spatial alignment with optical neural interfaces [12]. The latter points related to integration capabilities are of particular relevance since, in the era of multifunctional neural interfaces [13, 14], specific attention has been paid to the thermal effects of light delivery in different experimental configurations, including optogenetic activation/inhibition of neural activity, measurement of Raman scattering [15], and light‐induced dilation of cerebrovascular circuitry [16]. Numerous reports highlight how commonly used optogenetic illumination protocols easily generate temperature increases of the order of 0.2°C–2°C, with repercussions on spiking activity, which can be suppressed in multiple brain regions [17, 18]. For instance, Arias‐Gil et al. [19], offered a comprehensive optical thermography study of the thermal effect of superficial illumination from different laser intensities, wavelengths, and fiber sizes; Peixoto et al. [20], numerically modeled temperature effects induced by one‐photon light delivery from an optical fiber and the related influence on neuronal synchronization, while Picot et al. [21], studied the thermal effects of two‐photon (2P) illumination and proposed a holographic approach to minimize heating during 2P photostimulation. Furthermore, Owen et al. [17], demonstrated that deep brain illumination of the striatum in freely moving wild‐type mice produces a rotational bias despite the lack of opsin or fluorophore expression. However, most of the experimental approaches proposed so far to interface with intracortical and subcortical tissue require multiple implantable neural interfaces, each devoted to a specific physical channel (optical stimulation, electrical readout, and thermal readout), with an intrinsic offset between light delivery and temperature measurement volumes. A complementary approach proposed by Nam et al. [22, 23] utilizes a coaxial arrangement of an optical fiber and a tubular thermocouple sensor, which, although it offers a brilliant example of dense packing of the optical and thermal channels, doubles the implant cross section compared with the optical channel alone. Over the years, advances in microfabrication have enabled a number of temperature‐sensing probes with reduced cross section, such as microthermocouples, which shrink the detection volume down to near‐cellular scale [24, 25]. However, these probes typically require a separate optical channel to deliver light, so temperature sensing is not intrinsically coregistered with the region of optical excitation. Another important class of solutions is represented by all‐optical temperature readout based on fiber Bragg gratings (FBGs) written on the facet or along the core of polymer optical fibers [26, 27]. These probes enable fully optical temperature sensing along the waveguide, but the sensing region is located within the fiber rather than directly in the surrounding tissue, and they are generally not designed to deliver patterned illumination at the same location as the temperature measurement. In addition, most FBG‐based thermometry implementations rely on fiber encapsulations with diameters of a few hundred micrometers, which limits the minimum achievable implant cross section.

**FIGURE 1:**
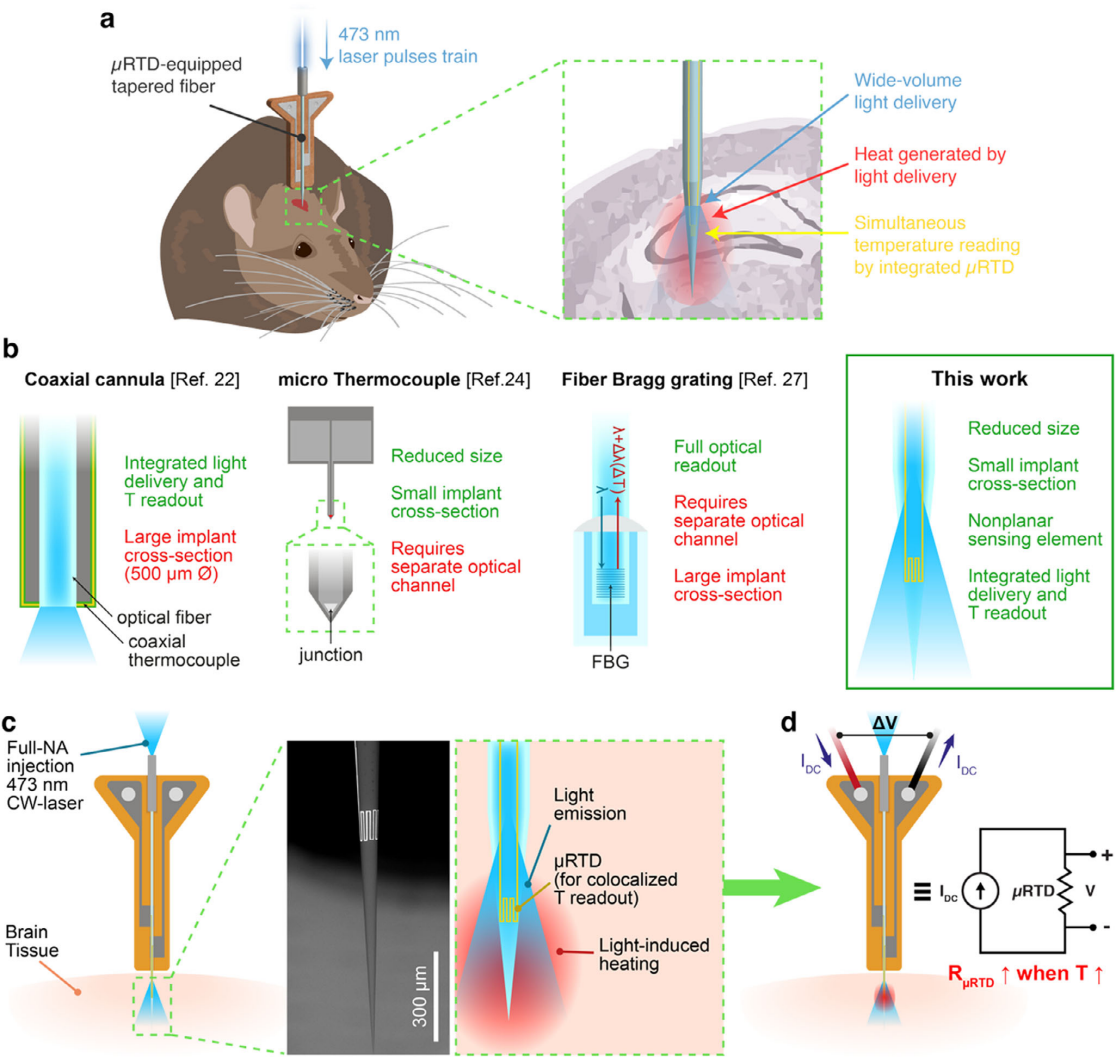
Concept and operating principle of the µRTD‐integrated tapered fiber neural interface. (a) Sketch of the target application of the presented device. The µRTD‐integrated TF is implanted in vivo with reduced implant cross section, offering a light delivery channel and simultaneous, co‐localized temperature detection. (b) Comparison between different temperature‐sensing neural interfaces. (c,d) Structural and functional schematics of the probe's optical delivery and thermal readout, respectively. The scanning electron micrograph shows a representative tapered fiber with non‐planar µRTD patterned on the taper.

In this work, to address the challenge of spatial mismatch and bulkiness in multifunctional neural interfaces at once, we engineered a compact, minimally invasive, integrated multifunctional implantable optical and thermal neural probe able to perform co‐localized temperature measurement and optical light delivery in deep brain regions in small rodents, with implant cross section of the order of a few microns (Figure 1a,b). The optical channel consists of a tapered optical fiber (TF) that, thanks to its modal properties, offers uniform light delivery over an extended area (Figure 1c) [28, 29], which can be further tailored to perform dynamically redirectable light coupling along its axis [30, 31]. The thermal sensing channel is directly integrated into the TF surface, and it consists of a non‐planar micro‐RTD (µRTD) element fabricated at the center of the light‐emitting region (Figure 1d), all around the fiber, patterning it over an angle of 360° through an unconventional additive manufacturing process based on Two‐Photon Polymerization (TPP) [32, 33]. First, we present an in vitro characterization of the probe's physical channels, demonstrating a linear response of the µRTD over a temperature range much larger than the range of interest for temperature variations in the brain. The effect of light delivery from the TF on the thermal sensing response was also quantified for several light delivery protocols characterized by pulse trains of different durations and light intensities. These were used to set up a simple method to eliminate the influence of photoelectric artifacts, relying on the separability of the frequency range of the temperature variations (on the scale of several seconds) and of the artifacts (locked with the laser pulses). The device was then tested in vivo, with the TF optical channel integrating the non‐planar µRTD used to deliver light in the mouse brain, applying illumination protocols commonly used in optogenetics and fiber photometry for multiple laser light intensities, while detecting the temperature variations at the exact same location. The tests enabled the quantification of photothermal effects in vivo, highlighting a mild influence of short stimuli on temperature, while 10‐s‐long pulses >10 mW/mm^2^ generated detectable temperature variations.

In this architecture, the µRTD relies on established biocompatible materials (Au as the resistive element, encapsulated in Parylene‐C, PrlC), and is realized through a TPP‐based non‐planar microfabrication process that patterns the metal track directly on the curved, tapered surface of the optical fiber. This strategy yields a conformal microscale sensor on a sub‐100‐µm‐diameter taper section with a length of the sensing element several times bigger than the radius of curvature, enabling co‐localization of optical delivery and thermal sensing within a minimal footprint, and is readily extendable to the integration of other microelectronic structures on 3D fiber‐based neural probes. For instance, we anticipate that the presented probe can be enriched with electrophysiology capabilities, as the same process has been used also to integrate microelectrodes on TFs in our previous work [34, 35]. We believe that the presented approach represents another arrow in the quiver of multifunctional neural interfaces.

## 2 Results and Discussion

### 2.1 Fabrication of the Temperature Sensor

**FIGURE 2:**
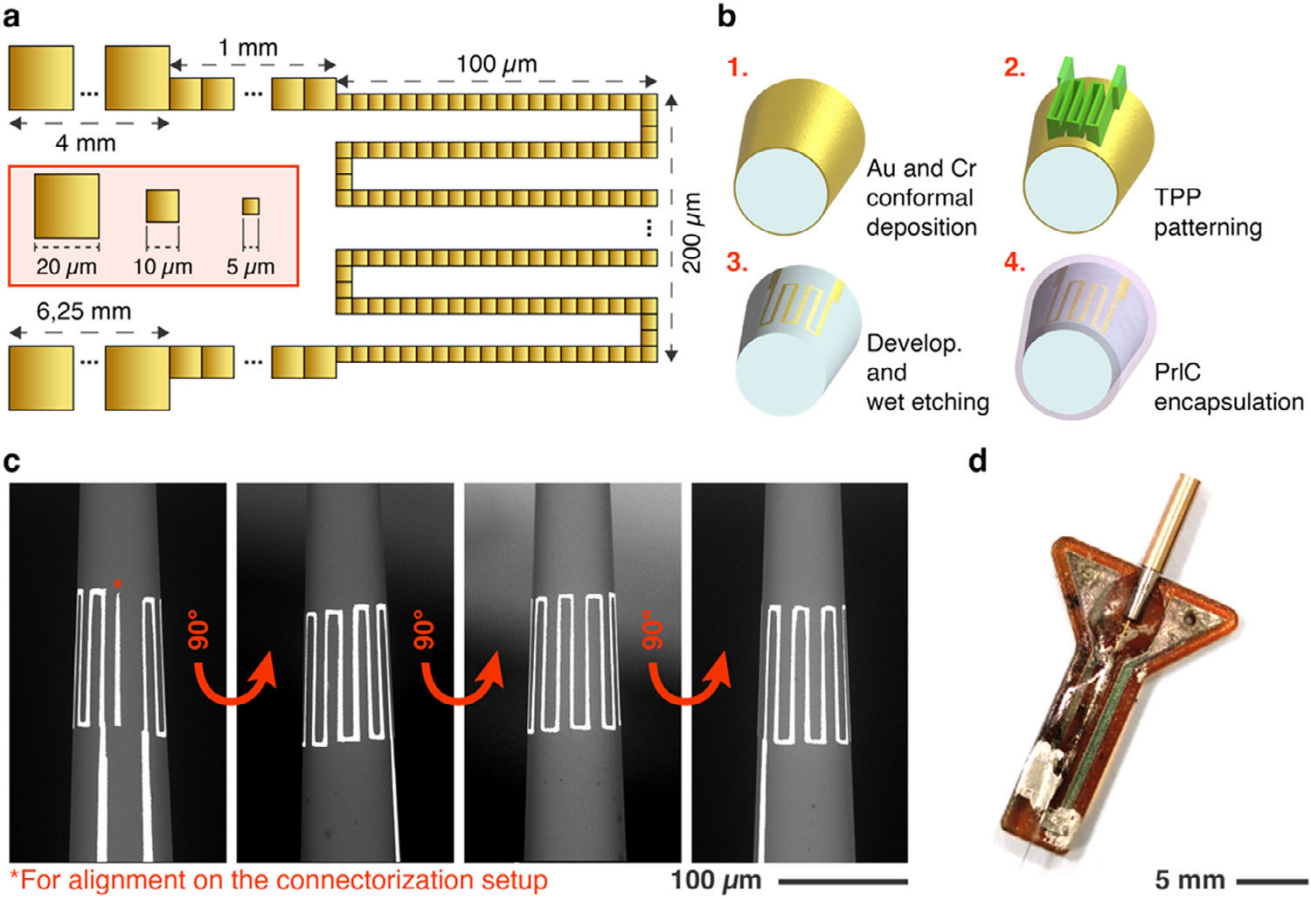
Design and fabrication of the µRTD integrated on a tapered optical fiber. (a) Scheme of the designed pattern, sketched as a series of small squares of the same thickness and with a resistance of 0.2 Ω. The total pattern comprises 964 squares. (b) Sketch of the principal steps of the fabrication process. The fiber is first coated with a 125 nm‐thick conformal layer of Au (plus Cr as an adhesion layer). A polymeric mask is built through TPP, and after a series of chemical steps the pattern designed in panel ‘a’ is impressed onto the fiber. The fiber is finally encapsulated by a 2 µm‐thick conformal layer of Prl‐C. (c) Scanning Electron Micrographs of the serpentine section of the realized pattern. The SEM image in each panel has been taken by rotating the fiber 90° around its axis with respect to the preceding panel. (d) Photograph of a representative complete device, with the fiber connected to a custom‐designed 3D printed PCB.

The design of the sensor takes into account two factors: on the one hand, the total footprint of the sensitive element must be smaller than the volume influenced by light delivery from the TF so that the probed region is uniformly affected by the potential heating effect of light on the surrounding neural tissue. On the other hand, the total length of the metal track must be long enough to demonstrate a reasonably high resistance value, allowing temperature variations of the order of 0.1 °C to be evaluated, this being a physiologically relevant temperature increase. Concerning the first consideration, the TFs we used in this work (fabricated from a 200/225 µm core/cladding diameter 0.22 numerical aperture fiber, with a taper angle of *φ *≅ 4°) feature an active region [36] – or the surface from which light is coupled with the external environment – that can be approximated with a conical surface with a height of *h* ∼ 1.25 mm and base diameter of *d* ∼ 85 µm, for a total surface of *S_taper_ *∼ 0.2 mm^2^. Therefore, the sensitive surface area was set to *S_sensor_* << *S_taper_*, on a footprint with *S_sensor_ *= 0.02 mm^2^. Moreover, in a compromise between ensuring even illumination around the µRTD and a surface area large enough to allow convenient fabrication, the active sensor was fabricated at the center of the active region, at a fiber diameter of *d* ∼ 60 µm. To meet the second design consideration, a standard serpentine design with a reduced transverse section with thickness of *t* = 125 nm was selected. Furthermore, the overall design, sketched in Figure 2a, was developed to consider also the dimensions of the custom‐printed Circuit Board (PCB) used to connect the sensor with an external acquisition/control system: it comprises a serpentine pattern with a width of 5 µm, a length of ∼1.46 mm, and a total footprint of ∼100 × 200 µm^2^, two 10‐µm‐thin, 1‐mm‐long tracks that start from the two ends of the serpentine, and two 20‐µm‐wide tracks, one 4‐mm‐long and the other 6.25‐mm‐long, which run along the fiber axis.

The pattern can be considered as composed of small squares of the same thickness connected in series, and its theoretical resistance value is given by the resistance of the single square multiplied by the total number of squares employed to form it. Au was selected as the material to fabricate the sensor, owing to its excellent stability and reliability over a wide temperature range and to its good electrical conductivity, allowing for efficient signal transmission. Hence, considering a value for the bulk resistivity of Au at room temperature [37] of *ρ_bulk,Au_*  =  2.44 × 10^−8^ Ω·m, the *sheet resistance* [38], or the resistance of a single square piece of thin material (▪) with contacts on two opposite sides of the square, is:

(1) $$R_{s}=\frac{\rho_{bulk,Au}}{t_{Au}}=\frac{2.44\times 10^{-8}\Omega \cdot m}{125\times 10^{-9}m}≃0.2\Omega /▪$$

Thus, the theoretical resistance of the designed metallic pattern resulted, according to Equation 1:

(2) $$R_{s}\cdot N_{squares}=0.2\frac{\Omega }{▪}\cdot 1005▪=201\Omega$$

The non‐planar temperature sensor integrated onto the TF was fabricated by adapting the TPP‐based process described in Refs [32, 35], which allows the manufacture of metallic patterns with custom‐defined geometries along and around the non‐planar TF surface (more details in *Experimental Section/Methods*). Figure 2b illustrates the main steps of the process: in brief, TPP was employed to create a polymeric mask projecting the chosen design on the non‐planar surface of an Au‐coated TF (with a thin Cr adhesion layer). The mask protected the underlying Au during subsequent wet etching steps and was then removed chemically. After removal of the mask, the patterned TF typically looks like the one shown in the scanning electron micrograph in Figure 2c.

After connecting the patterned TF with the custom PCB, the entire device was encapsulated by a conformal physical vapor deposition coating of PrlC, which electrically insulated the sensor from the external environment. PrlC is widely adopted in thermal sensing and actuation devices as an encapsulation layer [39, 40, 41, 42], and despite its low thermal conductivity *κ*  =  0.084 W/(m·K), previous studies have shown that for thicknesses on the order of 1 µm, it does not significantly affect the thermal response, while enhancing device stability and lifetime. Nonetheless, to estimate the thermal lag introduced by the PrlC layer, we applied a simple lumped thermal RC model, in which the PrlC layer is represented as a thermal resistance *R_th_  =  d/(κ·A)*, with *d*  =  2 µm being the PrlC thickness and *A* the total footprint area of the µRTD, and the gold sensing element as a thermal capacitance *C_Au_  =  ρ·c_p_·V*, where *ρ  =  19.3×10^3^* kg/m^3^ is the gold density, *c_p_*  =  129 J/(kg·K) its specific heat, and *V* ≈ 8.8×10^−16^ m^3^ the volume of the gold track, yielding a thermal lag time constant given by *τ  =  R_th_·C_Au_*. Based on this simple model, under the assumption of homogeneous, one‐dimensional heat flow across the PrlC layer and an effectively isothermal gold track, the thermal lag introduced by the PrlC coating is estimated to be τ ∼ 3 ms. This result indicates that the encapsulated µRTD retains a fast thermal response, with a time constant on the millisecond scale, making it suitable for dynamic temperature sensing in physiological environments. We emphasize that this simple RC model is used here solely to provide a conservative upper bound estimate of the intrinsic sensor time constant. The absolute temperature calibration and measurement accuracy are instead determined experimentally, as described in the following sections.

In Figure 2d, a photograph of the final device is displayed, while the principal steps of the fabrication process, along with typical images for each step, are presented in Figure S1. The pattern resistance was measured using a Source Measurement Unit (SMU, Keithley 2440) at a room temperature of *T* = 20 °C, resulting in *R_device_* = 195.4 ± 0.2 Ω. The measured value was slightly different from the one estimated in Equation 2 due to small differences introduced during manufacturing.

In a typical sensing configuration, the temperature sensor is driven by a direct current (DC), and the voltage generated between the two ends of the device is measured simultaneously by an SMU. However, applying a DC to the temperature sensor also results in the generation of heat by Joule effect (*self‐heating* from now on). The generation of an additional temperature gradient on the nearby tissue must be avoided. Hence, for sensing purposes, it was of primary importance to identify the highest DC value that generated a negligible self‐heating effect. For this reason, we developed a multiphysics finite element model (FEM) of the device to simulate this behavior numerically using COMSOL Multiphysics (details in *Experimental Section/Methods* and *Supporting Information*, simulation parameters in Table S1 and Table S2). At a steady state, the heat generated by the resistive element is dissipated to the surrounding environment in two ways: (i) on the upper surface the heat is taken by the PrlC layer which dissipates it in the air or water on top of it by the process of convection (ii) on the lower surface the heat conducts through the glass substrate and finally convects to the surrounding environment. In the model, the ambient environment temperature was set at 20 °C for air and 37 °C for a water environment to approximate the physiological temperature of the brain. Figure S2 reports the geometric structure of the numerical model and the temperature variation generated by self‐heating from the temperature sensor for different DC values, starting from the two aforementioned temperatures. The numerical results suggested that for a DC driving current of *I_DC_* = 0.1 mA the temperature variation for self‐heating in air is *ΔT_SH_* (*I_DC_*  =  0.1 mA) ≈ 0.2 °C, while in water it decreases to *ΔT_SH_* (*I_DC_* = 0.1 mA) < 0.1 °C, hence resulting in a negligible self‐heating contribution.

### 2.2 Characterization of the Temperature Sensor

**FIGURE 3:**
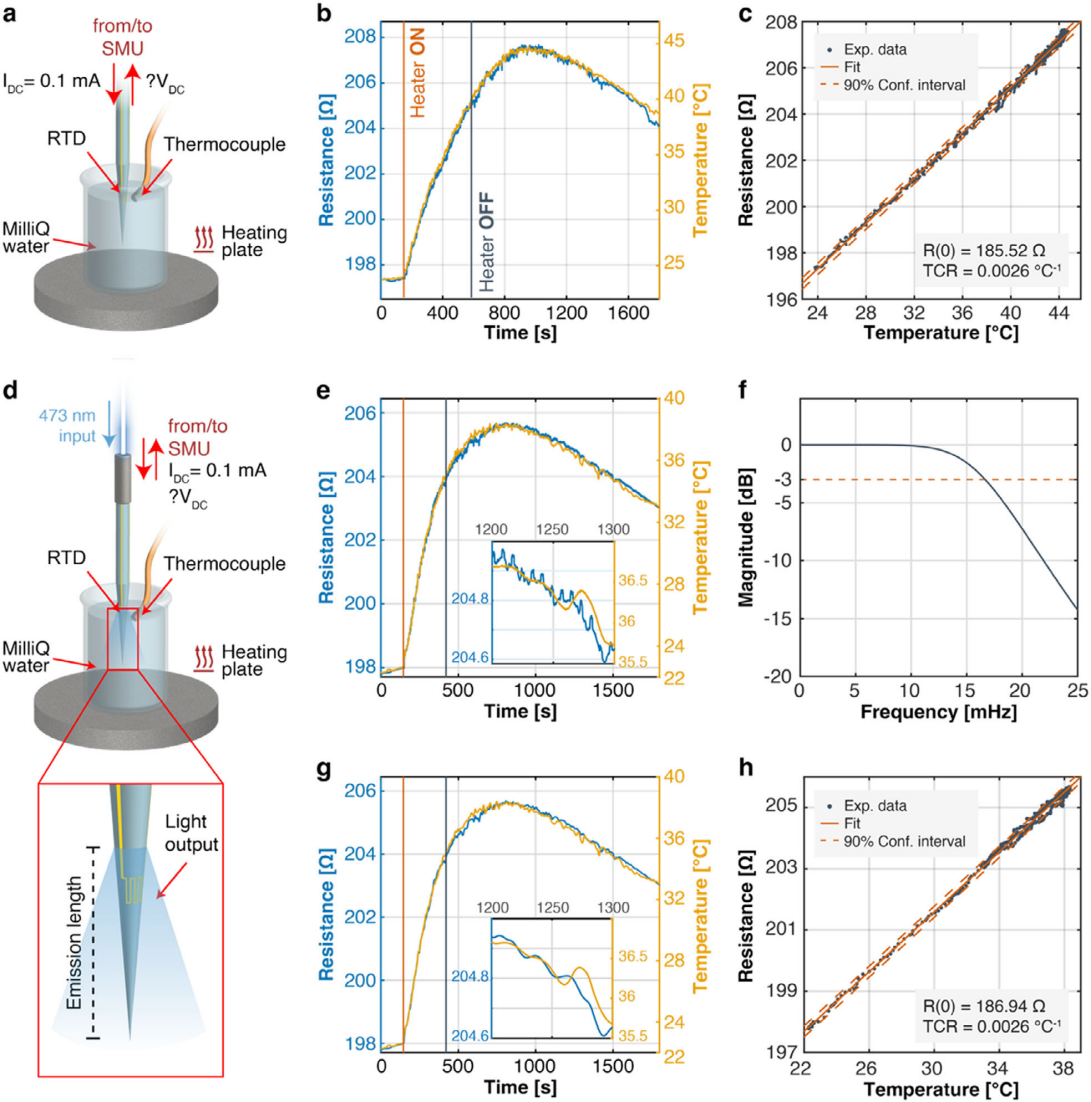
In vitro calibration of the µRTD and photoelectric artifacts filtering. (a) Sketch of the experimental setup employed for in vitro testing of the µRTD. (b) Raw data acquired with µRTD (blue line) and a commercial thermocouple (orange line) during heating ramp up and cooling down. The vertical lines indicate the time stamps at which the heater was turned on after 150 s (red line, labeled ‘Heater ON’), and at which it was turned off, after the commercial sensor detected a T = 40°C (blue‐gray line, labeled ‘Heater OFF’). (c) Resistance versus Temperature calibration curve extracted from the data in panel ‘b’. The dashed line represents the 90% confidence interval. (d) Sketch of the experimental setup employed for investigating the photoelectric artifacts. (e) Raw data acquired with µRTD (blue line) and a commercial thermocouple (orange line) while delivering a laser pulse train for the entire duration of the acquisition. The inset shows a zoom in on a 100 s‐long window, in which periodic peaks locked with the pulse train are clearly visible. (f) Transfer function of the low‐pass filter used remove photoelectric noise. (g) Filtered data obtained by applying the filter with the transfer function shown in panel ‘f’ to both the µRTD readings and the commercial thermocouple ones reported in panel ‘e’. (h) Final Resistance versus Temperature calibration curve. The dashed line represents the 90% confidence interval.

Before the in vivo experiments, the µRTD was calibrated in a Milli‐Q water bath. The experimental configuration is illustrated in Figure 3a. The µRTD‐integrated tapered optical fiber was submerged in a beaker filled with Milli‐Q water, placed on a hot plate to regulate the bath temperature. A thermocouple (TC), positioned in close proximity to the µRTD, served as a reference temperature sensor. An SMU supplied a constant 0.1 mA DC current to the µRTD and measured the resulting voltage drop across the sensitive element, enabling the extraction of the electrical resistance value *R*, which varies proportionally with temperature. After an initial thermal stabilization period of 150 s, the hot plate was activated. The temperature rose rapidly and reached the maximum recorded value after the heater was turned off, once the TC registered a temperature of 40°C. After reaching the maximum temperature, the system underwent a slow and nearly linear cooling phase. The raw *R* values are shown in Figure 3b (blue line) and compared with the TC readings (orange curve). The readings from the integrated µRTD closely tracked those of the thermocouple throughout the experiment, highlighting the reliability and stability of the µRTD under time‐varying thermal conditions.

These calibration data were used to estimate the Temperature Coefficient of Resistance (*TCR*) of the probe, which quantifies the relative change in electrical resistance of a conductor per degree Celsius of temperature variation, normalized to the resistance at a reference temperature of *T* = 0°C (i.e., *R_device_* (0)). The *TCR* was extracted by fitting the resistance versus temperature data reported in Figure 3c with the following equation, yielding minimal residuals (Figure S3) [43]:

(3) $$R_{device}\;\left(T\right)=R_{device}\;\left(0\right)\cdot \left[1+TCR\cdot T\right],$$

where *R_device_* (*T*) denotes the resistance of the µRTD at temperature *T*. The same calibration was carried out for *n *= 3 devices (resistance versus temperature data reported in Figure S4, full set of extracted parameters in Table S3), from which we extracted a *TCR* of 0.0025 ± 0.0001 °C^−1^ (mean ± SD, *n* = 3), which is consistent with values reported in the literature [43, 44], typically ranging from 0.0015 °C^−1^ to 0.0045 °C^−1^, and not far from the theoretical value of 0.0032°C^−1^. The resistance value at *T* = 0°C was found to be *R_device_* (0) = 184 ± 6 Ω (mean ± SD, *n* = 3). The *TCR*, in turn, defines the intrinsic sensitivity of the sensor, calculated as *S* = *TCR*·*R_device_* (0) ≅ 0.46 ± 0.02 Ω/°C (mean ± SD, *n* = 3). This quantity is set by the choice of sensing material (thin‐film Au) and by the serpentine geometry, which maximizes resistance within the ∼100 × 200 µm^2^ footprint on the taper, whereas the PrlC encapsulation and glass fiber mainly affect the thermal coupling and response time, but do not significantly alter the intrinsic sensitivity *S*. From these data, we estimated the measurement accuracy of the µRTD by computing the root mean square (*RMS*) of the difference between the readings of the TC and those of the µRTD after the calibration, over the same linear heating segment. This yielded an accuracy of *A* ≈ 0.25 ± 0.05 °C. These values indicate modest device‐to‐device variability (3%–4% for *R*(0), *TCR*, and *S*) and a consistent accuracy in the 0.2°C–0.3°C range.

The minimum resolvable temperature change is limited by the noise of the readout chain. For this reason, we quantified the noise for both the TC and the µRTD, which exhibited comparable noise levels. These were quantified by isolating the linear heating phase of the temperature ramp and applying a low‐pass Butterworth filter, with a cutoff frequency *f_c_* = 1/*τ_c_*, to separate signal (low‐frequency variations) and noise (higher‐frequency variations). We chose *τ_c_* = 30 s, a value justified below based on the spectral content of the in vivo temperature traces (see Section 2.3) and consistent with those used in previous studies of brain temperature monitoring [26]. The resulting *RMS* noise values were *RMS_TC_* ∼ 0.11 °C and *RMS_µRTD_* ∼ 0.12°C for the TC and the µRTD, respectively. After filtering out the high‐frequency electrical noise from both datasets (*τ_c_ *= 30 s), the accuracy improved to *A* ≅ 0.15 °C. The achievable accuracy can vary depending on the characteristic time scale of the thermal phenomena under investigation, as it depends on the acquisition time and corresponding noise level. This trade‐off is illustrated in Figure S5, displaying estimates of *A* as a function of *τ_c_* and *f_c_*, where the asymptotic convergence of *A* to ∼0.23°C can be observed.

The performance of the temperature sensor was further assessed under light illumination conditions, using a similar experimental approach while simultaneously delivering a train of laser pulses (*λ*  =  473 nm, frequency 40 Hz, duration 2 s, duty‐cycle 50%, inter‐burst interval 6 s) through the optical fiber, as sketched in Figure 3d. The laser wavelength and the pulse scheme were selected due to their widespread use in optogenetic stimulation paradigms. The laser power output was adjusted to yield a power density of 10 mW/mm^2^ at the taper surface, exceeding typical values used in optogenetics or fiber photometry experiments. Under these conditions, the measured µRTD resistance continued to closely track the reference TC readings, with the exception of a series of small and sharp periodic peaks precisely locked to the onset and offset of the light pulses, as shown in Figure 3e that were absent in the thermocouple readings. Their rapid rise and decay, with no measurable thermal lag, strongly suggests a photoelectric origin of the artifacts, rather than a photothermal effect, which would exhibit slower dynamics. The artifacts are likely caused by direct photon interaction with the µRTD, which is patterned directly onto the waveguide and thus exposed to both the evanescent field of guided modes and radiative leakage from the taper.

A characterization of the photoelectric artifacts as a function of the delivered power density is reported in Figure S6. Owing to their narrow frequency content and strict periodicity, these artifacts can be effectively removed by applying a low‐pass filter (example transfer function shown in Figure 3f). The resulting filtered resistance trace is shown in Figure 3g (filtering details are provided in the Experimental Section/Methods section and Supporting Information).

After filtering out the photoelectric artifacts, the resistance was plotted as a function of the temperature recorded by the thermocouple, yielding the calibration curve shown in Figure 3h. The extracted *R_device_* (0), *TCR*, sensitivity, and accuracy values (*R_device_* (0) ≅ 186.9 Ω, *TCR* ≅ 0.0026 °C^−1^, *S* ≅ 0.4885 Ω/°C, *A* ≅ 0.23 °C) were consistent with those obtained from the raw data acquired under dark conditions. Furthermore, it should be noted that the quantities obtained by analyzing the raw data without filtering the photoelectric artifacts differ from those reported for the filtered time trace by less than 0.1% (Figure S7), further supporting the robustness and reliability of the µRTD even under intense optical stimulation.

### 2.3 In Vivo Brain Temperature Measurement During Co‐Localized Light Delivery

**FIGURE 4:**
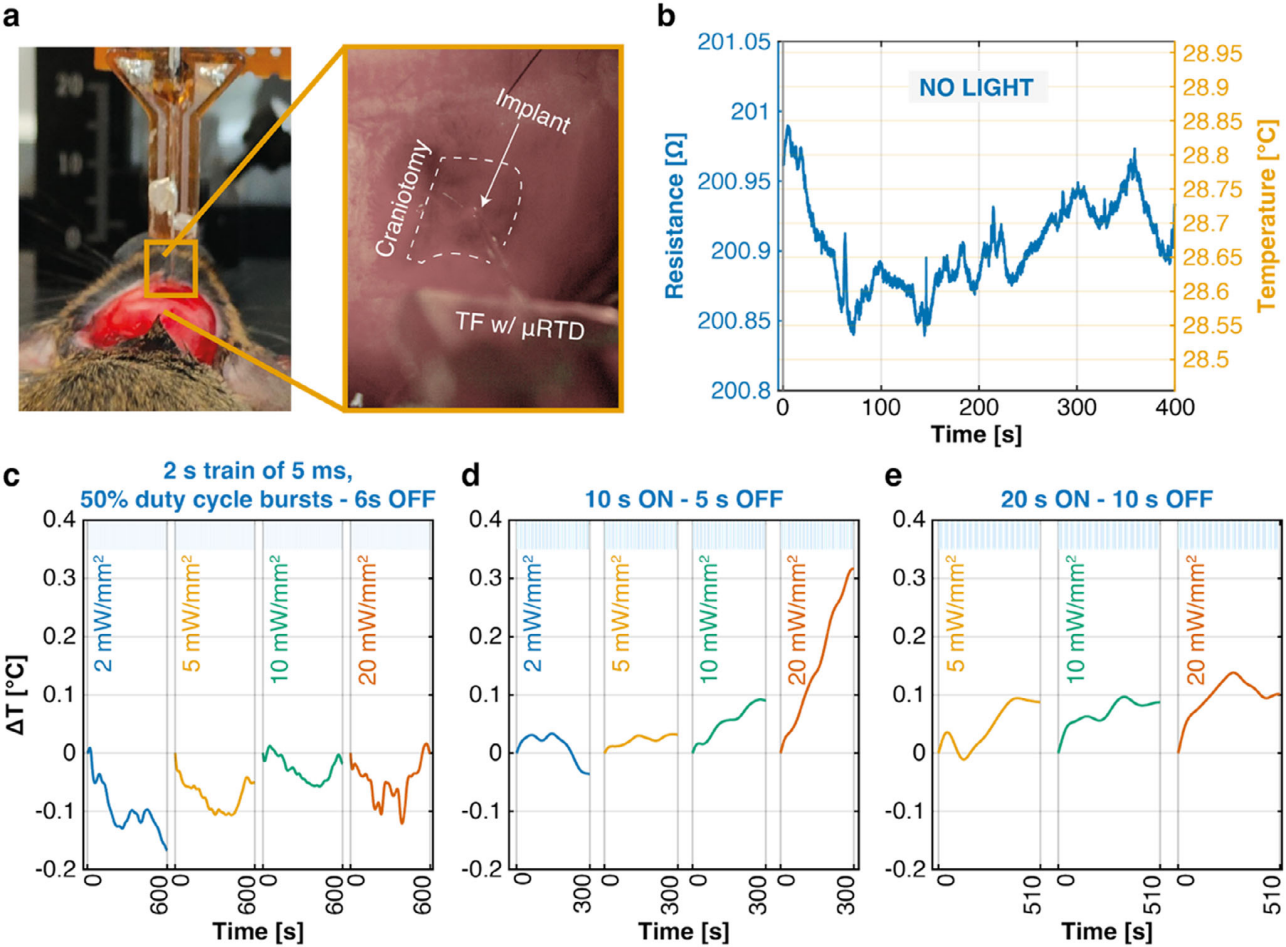
In vivo simultaneous light delivery and temperature monitoring. (a) Photograph of the µRT‐equipped TF implanted in C57BL/6 wild‐type mouse. The inset shows a magnified view of the implant region. (b) Detected temporal evolution of local tissue temperature in the mouse brain as a function of time with no light pulses applied. (c‐e) Variation of local brain temperature while delivering 473 nm light pulses through the tapered optical fiber hosting the temperature sensor. Measurements are reported for different ON and OFF periods and different power densities as labeled in the panels.

The integrated device for simultaneous optical neural interfacing and temperature detection was evaluated in vivo, in proof‐of‐concept experiments in the brain of a C57BL/6 mouse using the configuration shown in Figure 4a. The patterned optical fiber, bonded to the custom PCB, was implanted into the somatosensory cortex (details in Experimental Section/Methods), with the sensitive element positioned in cortical Layer 5. We selected this layer of the primary somatosensory cortex as a representative neocortical target because it is widely used in optogenetic experiments [45] and offers a large, superficial region that facilitates controlled implantation of the tapered fiber [46]. For a given wavelength, power density, and duty‐cycle, light‐induced heating is expected to be governed mainly by the optical and thermal properties of gray matter and by the light distribution from the optical fiber, so qualitatively similar subdegree temperature changes are anticipated in other gray matter targets under comparable stimulation conditions [18]. All in vivo proof‐of‐concept measurements were performed in acute preparations under anesthesia, and the present work does not constitute a systematic chronic implantation study.

Following the implantation and a rest period of 15 min, a constant DC current of 0.1 mA was supplied to the µRTD while the voltage drop across the device was continuously recorded at a sampling rate of 10 Hz. Local temperature was then estimated using the calibration curve described in the previous section. A representative measurement trace of brain temperature over ∼400 s is reported in Figure 4b, showing spontaneous fluctuations on the order of 0.3°C, corresponding to resistance variations in the range of 200.8–201 Ω.

To assess the effect of light absorption on local brain tissue temperature, we then investigated three distinct illumination protocols: (i) an optogenetics‐inspired pulse train with frequency of 40 Hz, duration of 2 s, duty‐cycle of 50%, and inter‐burst interval of 6 s; (ii) continuous ‘short interval’ illumination with 10‐s‐long light exposure and 5 s off‐time duration; (iii) continuous ‘long interval’ illumination with 20‐s‐long light exposure and 10 s off‐time duration, chosen in order to maintain the same duty‐cycle as in the short interval protocol. In all cases, illumination was delivered in repeated ON/OFF cycles over several minutes. The first paradigm reflects a standard light stimulation protocol for eliciting neural activity in optogenetics experiments [28], while the latter two are representative of protocols used for optogenetic inhibition [47, 48], and in vivo Raman spectroscopy [15]. Furthermore, the choice of 10 and 20 s ON periods is consistent with previous work [18] on light‐induced brain heating in mouse cortex, which indicates that at depths comparable to those probed in this work, the temperature approaches its steady‐state value within ∼5–15 s after light onset, with only modest additional increases for longer illumination.

The acquired data at different light power densities are reported in Figure 4c–e, displaying the measured temperature variations after filtering out the photoelectric artifacts (raw data with artifacts are reported in Figure S8). To quantify the effect of the filtering and to justify the choice of *τ_c_*, we analyzed the frequency content of representative in vivo traces for the optogenetics‐like and for the 10 s ON/5 s OFF protocols at 20 mW/mm^2^ (Figure S9). The power spectral density (PSD) of the raw signals shows that the vast majority of the energy associated with the slow temperature dynamics is confined below 0.01 Hz, whereas the photoelectric artifacts appear as narrow spectral lines at frequencies ≥0.12 Hz and higher harmonics. Applying a fourth‐order Butterworth low‐pass filter with *τ_c_* = 30 s (*f_c_* = 1/*τ_c_* ≈ 0.033 Hz) strongly attenuates these high‐frequency components while leaving the <0.01 Hz band essentially unaffected, as confirmed by the overlap between the PSDs of raw and filtered signals in this range. The corresponding group‐delay curves indicate that at 0.01 Hz the filter introduces a delay of <7 s (around 7% of the ∼100 s period of the temperature transients), so filtering results only in a minor horizontal shift without appreciable distortion of the thermal amplitude. Together, these analyses demonstrate that the designed filter efficiently suppresses high‐frequency photoelectric artifacts while preserving the slow, physiologically relevant temperature changes reported in Figure 4c–e. For the optogenetics‐like illumination (Figure 4c), no temperature change correlated with light delivery was detected, even at the highest investigated power density, with the µRTD readings remaining within the range of physiological oscillations observed in the absence of illumination (Figure 4b). In contrast, clear temperature rises were recorded during the 10 s ON/5 s OFF protocol (Figure 4d), where the local temperature progressively increased over repeated cycles, reaching ∼0.1 °C at 10 mW/mm^2^ and ∼0.3 °C at 20 mW/mm^2^ after 20 cycles. A minor but detectable increase of temperature (<0.05 °C after 20 periods) was already observable at 5 mW/mm^2^. When the off interval of the laser was extended to 10 s while maintaining the duty‐cycle (20 s light ON/10 s OFF, Figure 4e), the traces at 10 mW/mm^2^ and 20 mW/mm^2^ displayed only a slight initial temperature increase during the first cycles of the illumination protocol, superimposed on other fluctuations. This behavior likely reflects a balance between light‐induced heating and intrinsic brain cooling mechanisms [49, 50], such as blood perfusion, which efficiently remove heat from the stimulated region. At 5 mW/mm^2^, no consistent correlation between light onset and temperature change was observed.

To relate these in vivo temperature changes to existing quantitative models of light‐induced brain heating, we compared our data with the predictions of Stujenske et al. [18], who combined Monte Carlo light transport with a bioheat model and in vivo validation for 473 nm light delivered through 200‐µm‐diameter flat‐tip optical fibers. For a fixed geometry and wavelength, their results indicate that the peak temperature increase scales approximately linearly with an effective optical power *P_eff_* = *P × D*, defined as the product of total power and duty‐cycle. From their reported data, an empirical slope of ≈0.1–0.2 °C per mW of effective power can be extracted in the hottest region near the fiber tip. Applying this power–duty‐cycle scaling to our stimulation parameters, the total powers corresponding to 5, 10, and 20 mW/mm^2^ at the taper surface (≈1, 2, and 4 mW, respectively) and the duty‐cycles used in vivo (50% for the optogenetics‐like trains and 2/3 for the longer ON/OFF protocols) are expected to produce sub‐degree temperature rises of ≈0.1°C–0.5°C around a flat‐tip fiber. The temperature changes measured with the integrated µRTD are slightly lower but of the same order of magnitude, an observation that is consistent with the different optical geometry: in the Stujenske configuration, the same total power is injected from a compact circular facet, leading to relatively high local power densities and a larger peak *ΔT* near the fiber tip, whereas the TF distributes light over a larger conical surface and a longer axial distance, spreading the deposited energy over a larger tissue volume and thereby reducing the local maximum temperature increase. Because the literature model assumes a compact flat‐tip geometry, using its predictions as a reference for our extended tapered device is therefore conservative and supports the conclusion that the tested stimulation paradigms operate well within the subdegree heating regime.

Overall, the reported proof‐of‐concept in vivo results suggest that the integrated probe can resolve subtle, light‐induced thermal dynamics in the brain, under stimulation protocols that bring tissue temperature close to its steady‐state increase within tens of seconds, offering a powerful tool to characterize heating effects across different stimulation paradigms.

## 3 Conclusion

The integration of a non‐planar µRTD onto the surface of a TF presented in this work, introduces a novel, compact, and multifunctional platform for the co‐localized delivery of light and temperature monitoring within the brain. This configuration overcomes key limitations of conventional neural interfaces, where the physical separation between stimulation and sensing elements can result in spatial mismatches and reduced precision. By directly fabricating the µRTD via TPP onto the curved surface of the TF, precise spatial co‐localization between the optical and thermal modalities is achieved, enabling accurate real‐time measurement of temperature changes exactly in the region exposed to optical stimulation.

This integration is motivated by the growing interest of the scientific community in characterizing photothermal effects generated during optogenetic excitation and inhibition of neural activity, in vivo Raman spectroscopy as well as the emerging neuromodulation techniques based on thermoplasmonic heating. Several studies have demonstrated that light intensities typically used in these applications can elevate tissue temperature by 0.2°C–2°C, a variation sufficient to modulate neuronal excitability independently from the expression of optogenetic actuators. The ability to monitor such temperature changes in real‐time, and at the exact stimulation site, is thus essential not only for basic neurophysiological studies but also for the development of safe and effective neuromodulation therapies. From a fabrication standpoint, the use of TPP to create high‐resolution polymer masks for metal patterning on non‐planar substrates represents a significant advancement. This approach enabled the definition of a gold serpentine pattern with precise control over the geometry, conforming directly to the curved surface of TFs. The serpentine layout was designed to maximize resistance within a compact footprint (∼100 × 200 µm^2^), thereby enabling high thermal sensitivity while maintaining a compact form factor. Gold was selected as the sensing material for its chemical stability, biocompatibility, and well‐established electrical and thermal properties, further contributing to the reliability and reproducibility of the µRTD.

Electrical characterization of the fabricated devices yielded a resistance of approximately 195 Ω, matching theoretical predictions and confirming the fidelity of the patterning process. To evaluate potential self‐heating effects under DC bias, an inherent challenge in resistance‐based sensors, finite‐element modeling was performed. Simulations indicated that driving currents below 0.1 mA result in negligible temperature rise (<0.1 °C), validating the thermal safety of the readout conditions used during experiments. In vitro calibration in an aqueous bath demonstrated a linear response of the µRTD over the physiologically relevant temperature range, with a *TCR* of 0.0025 ± 0.0001 °C^−^^1^. The performance metrics extracted from the µRTD‐TF device (*TCR* = 0.0025 ± 0.0001 °C^−^^1^, intrinsic sensitivity *S* ≈ 0.46 ± 0.02 Ω/°C, *A* ≈ 0.25 ± 0.05 °C) are comparable to those reported for thin‐film Au RTDs [51], and for commercial Pt intracranial temperature probes using PT100 RTD sensors [52], which typically exhibit *TCR* values in the range 0.002–0.0039 °C^−^^1^ and specified accuracies on the order of 0.1°C–0.3°C. The benefit of the present approach therefore lies not in a fundamentally higher sensitivity, but in achieving RTD‐class performance in a non‐planar geometry directly integrated on a tapered optical fiber, which simultaneously minimizes the implant cross‐section and coregisters temperature sensing with the region of optical emission in brain tissue.

The direct integration of the µRTD onto the TF introduced photoelectric artifacts, attributed to the interaction of light guided through the fiber with the metallic structure. Importantly, in the probe design, no shielding layers able to abate photoelectric artifacts, as demonstrated in our previous work [35], was incorporated. This was done deliberately, with the aim of having the µRTD fully immersed into the tissue volume subjected to illumination. As a result, the detected artifacts appeared in the resistance traces as periodic signal fluctuations components, synchronously locked to the light pulses. However, by exploiting the temporal separation between high‐frequency light‐induced artifacts and low‐frequency thermal transients, they were readily mitigated via low‐pass filtering, enabling clean thermal readout without significant post‐processing complexity.

The proof‐of‐concept in vivo experiments conducted in the mouse brain highlight the practical utility of the proposed system under realistic neurobiological settings. By enabling simultaneous light delivery and localized temperature variations monitoring through a single probe, we were able to directly evaluate photothermal effects during optogenetics‐like stimulation protocols. For short light pulses of 5 ms (duty‐cycle 50%), commonly used in neural activation paradigms, no measurable temperature change was observed, even at relatively high power densities. This observation supports the assumption that such brief stimulation protocols are thermally safe when performed within standard intensity ranges. In contrast, prolonged light exposure (10 s at 10–20 mW/mm^2^) resulted in a clear rise in local temperature, underscoring the risk of thermal accumulation during sustained illumination. These results are consistent with previous simulation and experimental studies, reinforcing the importance of real‐time thermal feedback in experimental designs involving prolonged or high‐intensity optical stimulation. Indeed, from a biophysical standpoint, for a given wavelength, power density and duty‐cycle, light‐induced temperature changes are primarily determined by the optical and thermal properties of gray matter and by the light distribution from the optical fiber, as described in standard light–heat and bioheat models of brain tissue [18, 50]. These models treat gray matter as a continuum characterized by absorption, scattering, conductivity, and perfusion, and show that blood flow mainly shapes the baseline temperature and the spatial‐temporal spread of heating rather than introducing strong area‐specific differences at comparable depths. Therefore, we expect qualitatively similar temperature rises in other gray matter targets (e.g., motor cortex, hippocampal subregions, thalamic nuclei) when using comparable stimulation parameters, even though the exact temporal profile may vary.

Beyond conventional optogenetics, the presented integrated opto‐thermal probe provides unique opportunities for applications that require closed‐loop neural stimulation paradigms. For instance, it can function as a real‐time thermal feedback channel in studies exploring the rising field of upconversion nanoparticles, materials that absorb near‐infrared light and emit visible radiation, that often induce unwanted and difficult‐to‐quantify heating of surrounding tissue [53, 54]. Likewise, the probe is suited for emerging approaches based on thermoplasmonic heating and near‐infrared absorption, which enable modulation of neural activity without the need for genetic targeting [55, 56].

One of the most compelling aspects of this technology lies in its extensibility and integrative potential. The additive nature of the TPP process allows the fabrication of additional functional elements, such as microelectrodes for electrical recording, directly onto the same fiber. Previous work by our group has demonstrated the feasibility of such integration. Moreover, the surface of the TF could be decorated with gold nanoislands or other plasmonic nanostructures to enable active heating via thermoplasmonic effects [57, 58, 59], paving the way for hybrid stimulation strategies that combine optical and thermal modalities. A further advantage of the presented platform is its reduced invasiveness. By maintaining the cross‐section of the implant close to that of the original optical fiber, the system minimizes tissue damage upon insertion and reduces the foreign body response, which is particularly critical for chronic implantation scenarios [29]. This contrasts with other dual‐function probes that rely on coaxial or side‐by‐side arrangements of light delivery and sensing components, often at the cost of significantly increased implant size.

Despite the strengths of the presented approach, several aspects remain to be addressed in future developments. This study primarily focuses on the integration of the µRTD; however, off‐board circuitry, such as Wheatstone bridge configurations, could be implemented to further enhance measurement sensitivity and robustness. Long‐term stability and biocompatibility of the device should be systematically investigated, particularly under chronic implantation conditions, with specific attention to the role of the outer insulating layer that directly interfaces with neural tissue [60]. Closely related to this, time‐dependent degradation of the encapsulation and interfacial mechanics at the polymer/metal and polymer/substrate interfaces under physiological conditions are critical considerations for long‐term performance and reliability: mechanical loading, viscoelastic creep, and thermally induced strains can give rise to interfacial shear stresses, microcrack nucleation, and progressive debonding. The sensing element and interconnects are made of gold and are encapsulated in PrlC on a glass fiber substrate, a material stack that is widely used in implantable neural probes and other active biomedical devices and is known to exhibit good biocompatibility and barrier properties over chronic timescales [61, 62, 63]. Nevertheless, in our non‐planar, tapered geometry the Au/PrlC and PrlC/glass interfaces may be subjected to mechanical and thermomechanical stresses under chronic implantation, which can, over long durations, promote local delamination, moisture ingress along interfacial defects, and changes in the effective thermal and electrical pathways of the µRTD, ultimately manifesting as drift in the *R*(*T*) characteristic or encapsulation failure [64, 65]. The proof‐of‐concept in vivo experiments presented here were carried out in acute preparations, and a comprehensive assessment of long‐term stability and tissue response will therefore require dedicated future studies (i.e., accelerated aging and soak tests, repeated in vitro calibrations, and chronic implants with histological evaluation).

Furthermore, the current system relies on wired connections, which may constrain its applicability in freely moving animals. From a system perspective, the µRTD is well suited for wireless integration because it is a passive, low‐power resistive sensor that can be biased with a low DC current and read out using a compact head‐mounted or fully implantable wireless module, similar to those used in recent wireless optogenetic and neuromodulation systems [66, 67, 68]. Importantly, because the temperature dynamics associated with light‐induced heating occur at low bandwidth (well below spiking), sampling and telemetry requirements are modest. A practical configuration would retain the implanted tapered fiber with the integrated µRTD, while routing the proximal ferrule and µRTD leads to a miniaturized wireless front‐end. Wireless light delivery through tapered fibers has already been demonstrated using head‐mounted laser‐diode coupling architectures [69], supporting feasibility for our platform. With respect to current budget, a typical µRTD bias would add only a small incremental draw compared with the optical drive currents typically required for wireless optogenetic stimulation, in addition to the current required by telemetry and control electronics. While a fully integrated wireless system is beyond the scope of the present work, achieving untethered operation would primarily require system‐level integration and packaging of the multifunctional probe with wireless optical coupling and telemetry, enabling a broader spectrum of behavioral experiments and applications.

In conclusion, the multifunctional probe presented here constitutes a robust and adaptable tool for precise thermal monitoring in optically stimulated neural tissue. By enabling real‐time, spatially resolved temperature feedback at the stimulation site, it improves the reliability and interpretability of optogenetic experiments. Through the use of advanced fabrication strategies and a modular design approach, this platform opens the door to next‐generation neural interfaces with integrated sensing, stimulation, and closed‐loop feedback capabilities.

## 4 Experimental Section/Methods

### 4.1 Fabrication of Devices

Tapered fibers were obtained starting from Thorlabs FG200LEA commercial multimode fibers (0.22 numerical aperture, pure silica core, fluorine‐doped silica cladding, 200/225 µm core/cladding diameter), tapered using a (Sutter Instruments Co. P‐2000). Conformal Cr (Sigma Aldrich cat. no. 3748490) and Au (Kurt J. Lesker cat. no. EVMAU40SHOT) deposition were performed using an electron beam evaporator (Thermoionics Laboratory Inc.). The µRTD patterning was performed using the custom TPP setup described in Ref. [70]. After chemical wet etching (Cr etchant Sigma Aldrich cat. no. 651826 and Au etchant Sigma Aldrich cat. no. 651818) and photoresist mask removal (via Piranha solution 3:1, H_2_SO_4_ Sigma Aldrich cat. no. 339741 and H_2_O_2_ Sigma Aldrich cat. no. H1009), the patterned fiber was connected to a custom PCB, printed using a multi‐material, multi‐layer high‐resolution nanocomposite‐based 3D printer (Nano Dimension Dragonfly IV), manually with silver paste (RS Pro Conductive Paint cat. no. 186–3600). Subsequently, PrlC conformal coating was performed using a Parylene Coater (Specialty Coating Systems PDS‐2010) and Parylene‐C dimer (Specialty Coating Systems). Interfacing with external driving/acquisition hardware was achieved by soldering two insulated copper wires (Tefzel Insulated 30AWG wire) connected to two 0.5 mm diameter pins at the two commection pads of the PCB, using a tin welder (Weller WSD 81) and soldering alloy (Loctite C 502 97SC 5C 0.25 mm G).

### 4.2 Characterization Measurements

Full‐NA light injection was performed using a custom build setup. A 473 nm CW laser (Hübner Photonics Cobolt 06‐MLD) was expanded 10 times in diameter by a couple of plano‐convex lenses in a beam expander configuration. The expanded beam was routed towards the back aperture of an objective lens (Nikon CFI Plan Fluor 10x) and coupled to an SMA‐ferrule patch‐cord made by the same fiber used for the TF. The power density and the temporal shape of the pulse trains were controlled by digitally driving the CW laser, BNC‐connected to a National Instruments DAQ 6363, with a custom written Python code. The µRTD values were collected using a Keithley 2440‐C SMU, connected to a computer using a National Instruments GPIB‐USB‐HS controller, with a custom written Python code.

### 4.3 In Vivo Experiments

The experimental manipulations on mice described in this paper are performed in accordance with the Italian Ministry of Health under the ‘protocol no. 429/2017‐PR’ approved by the Italian Ministry of Health. A C57BL/6 wild‐type mouse was anesthetized with Avertin (a preparation of tribromoethanol and tert‐amyl alcohol) and craniotomized under a stereotaxic apparatus. After surgical removal of the scalp the dura mater was also removed and somatosensory cortex was exposed. The device was implanted at coordinates −1.5 mm anteroposterior, 2.7 mm mediolateral from Bregma. The implant depth was chosen in order to place the sensitive element in layer 5 of the somatosensory cortex, hence positioning the fiber tip at dorsoventral depth of 1.3 mm. The device was fixed to a piezoelectric z‐motor and the implant was performed at a constant speed of 1um/sec to minimize neural damage. Light stimuli were applied using the same setup described in Experimental Section/Methods section “Characterization measurements”.

### 4.4 Data Analysis

Extraction of noise levels for the µRTD and the reference thermocouple was performed in Mathworks MATLAB starting from the data presented in Figure 3b. The two raw signals are hereafter referred to as RTD(t) and TC(t). A fourth‐order low‐pass Butterworth (cut‐off frequency f_c_ = 1/τ_c_, τ_c_ = 30 s) was applied to RTD(t) and TC(t), obtaining the extracted signals s_RTD_(t) and s_TC_(t). Noise traces were extracted as n_RTD_(t) = RTD(t)—s_RTD_(t) and n_TC_(t) = TC(t)—s_TC_(t) and used to calculate the RMS values in linear heating phase given in the main text.

Accuracy was calculated as the RMS value of the difference between the raw signals A_raw_ = RMS[RTD(t)—TC(t)] ≅ 0.23°C and after noise removal A_denoise_ = RMS[s_RTD_(t)—s_TC_(t)] ∼ 0.16. This latter is depicted for multiple values of f_c_ and τ_c_ in Figure S5.

## Funding

European Union's Horizon 2020 Research and Innovation programme under the Marie Sklodowska‐Curie Action grant agreement (#101106602). European Union's Horizon 2020 Research and Innovation Program (#101016787). European Union's Horizon 2020 Research and Innovation Program (#828972). NextGenerationEU PNRR MUR – M4C2 – Investimento 1.5 – Avviso “Ecosistemi dell'Innovazione” CUP J33C22001220001 and Novo Nordisk Foundation (#NNF23OC0079433), European Union ‐ European Research Council (#101125498)

## Conflicts of Interest

M. De Vittorio and F. Pisanello are founders and hold private equity in Optogenix, a company that develops, produces, and sells technologies to deliver light into the brain. The other authors do not declare any competing interests.

## Supporting information




**Supporting File**: adma72918‐sup‐0001‐SuppMat.docx

## Data Availability

The data that support the findings of this study are available from the corresponding author upon reasonable request.
