# Supplementary material for: Localized Temperature Monitoring in Mouse Brain during Light Delivery via a Non‐Planar Tapered Fiber‐Integrated µRTD Sensor
Source: Adv Mater. 2026 Apr 2;38(41):e19655. doi: 10.1002/adma.202519655 (PMC13393975; doi:10.1002/adma.202519655)

Supporting Information

Localized Temperature Monitoring in Mouse Brain during Light Delivery via a Non-Planar Tapered Fiber-Integrated µRTD Sensor

Antonio Balena*, Marco Bianco, Barbara Spagnolo, Muhammad Fayyaz Kashif, Alberto Bramati, Massimo De Vittorio, Ferruccio Pisanello*

## **Fabrication Process**

**Figure S1.** **Steps of the fabrication process**, along with typical images for each step


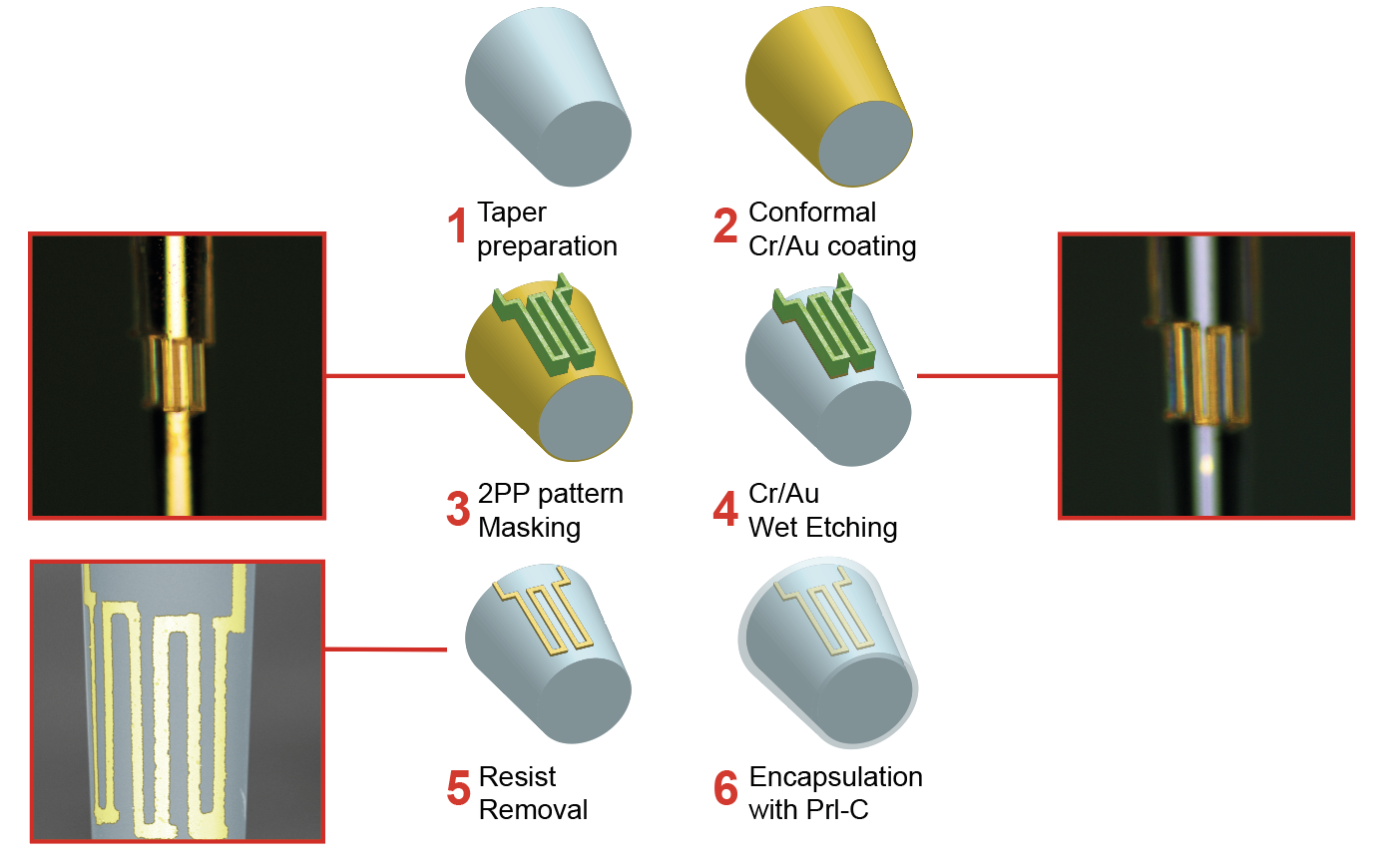


## **Numerical Modeling of the RTD and self-heating estimation**

The simulations considered a layered structure, reported in **Figure S2a**, which follows the geometrical and material characteristics of the fabricated device, described in the main text. The device consists of a gold resistive element with thickness $t_{Au}=125 nm,$ placed on a 60 µm-thick glass substrate (equivalent to the fiber diameter at which the resistor was fabricated). Two long tracks start from the two ends of the resistive element, and each terminates at a contact pad. The tracks, as well as the resistive element and the connector pads, are also made of gold. A thin layer of Parylene-C covers the device from the top to avoid polarization of the electrolyte due to the DC injected into the resistive element and the electrical tracks. The geometrical parameters of the different components of the device used in the simulation model are reported in **Table S1**. As shown in **Figure S2b**, in which the resulting self-heating heatmap is reported, the effect is mainly condensed in correspondence with the serpentine. Hence, to reduce the computational resources demand, the tracks length has been reduced to 200 µm.

**Table S1.** Geometrical parameters of the device components.

| Object | Length (µm) | Width (µm) | Thickness (µm) |
| --- | --- | --- | --- |
| Glass substrate | 440 | 180 | 60 |
| Gold circuit | 700 | 5 | 0.125 |
| Parylene C layer | 440 | 180 | 1 |
| Tracks | 200 | 5 | 0.125 |
| Contact pad | 60 | 60 | 0.125 |

The multiphysics model simulates the electrical heat generation and the heat transfer characteristics of the device, in each of its components. In particular, within COMSOL Multiphysics, the Heat Transfer in Solids Interface of the Heat Transfer Module in combination with the Electric Currents from the AC/DC Module were employed. A DC current is sent through the resistive circuit element by setting one of the contact pads as the terminal node boundary and keeping the other contact pad as the ground boundary. The electrical current generates heat due to the resistive losses known as the Joule heating effect. The produced volumetric heat power density inside the resistive element is given by:

$Q_{dc}= \vec{J}\cdot\vec{E}=\sigma\left| \nabla V \right|^{2}$ in $W/m^{3}$.

The absolute temperature is calculated employing the general heat diffusion equation:

$$\rho C_{p}\frac{\partial T}{\partial t} =\kappa\nabla^{2}T+\sigma\left| \nabla V \right|^{2},$$

where $\rho$and $C_{p}$ are the density and specific heat at constant pressure and $\kappa$is the thermal conductivity of the surrounding medium. The Joule heating effect (resistive losses) is given by the term $\sigma\left| \nabla V \right|^{2}$ where $\sigma$is the electrical conductivity of the resistive element and $V$is the electrical potential induced by the DC current. The details of the material properties used in the model are summarized in **Table S2**. For glass and gold, the material properties are taken from COMSOL library while for Parylene C the data is taken from Ref.^[6]^.

**Table S2.** Details of the material properties used in the model (glass and gold parameters are integrated in Comsol packages, while Parylene-C parameters are taken from Ref 6.

| Material | $\sigma[S/m]$ | $\kappa[W/(m\cdot K)]$ | $\rho[kg/m^{3}]$ | $C_{p} [J/(kg\cdot K)]$ |
| --- | --- | --- | --- | --- |
| Glass | 1⨯10^-14^ | 1.38 | 2203 | 703 |
| Gold | 4.11⨯10^7^ | 314 | 19320 | 125.604 |
| Parylene C | - | 1000 | 1289 | 3500 |

At a steady state, the heat generated by the resistive element is dissipated to the surrounding environment in two ways. (i) On the upper surface the heat is taken by the Parylene-C layer which dissipates it in the air or water on top of it by the process of convection (ii) on the lower surface the heat conducts through the glass substrate and finally convicts to the surrounding environment. In the model, the ambient environment temperature was set at 20 °C for air and 37 °C for water environment to approximate brain physiological temperature. The heat transfer coefficients of $h=5 W/(m^{2}K)$ and $h=20 W/(m^{2}K)$for the air and water surrounding environments, respectively, were used at the heat flux boundary. Insulation boundary condition was used at the sides of the glass substrate.

**Figure S2c** reports the temperature variation generated by self-heating from the thermistor for different DC current values at the two starting temperatures. From these simulated data, we observed that for a DC driving current of $I_{DC}=0.1 mA$ the temperature variation for self-heating is $\Delta T_{sh}\left( I_{DC}=0.1mA \right)<0.1^{\circ}C$, hence resulting in a negligible self-heating contribution.

**Figure S2. (a)** A schematic illustration of the device used in the simulation model. The materials and their respective thicknesses are shown in the 1D inset. **(b)** A representative heat map of the calculated temperature profile of the resistive device. **(c)** The calculated maximum temperature due to self-heating, which corresponds to the region around the resistive element, is shown as a function of the input DC current. The upper panel shows the results for air whereas the lower panel shows the results for the water as surrounding environment.

***
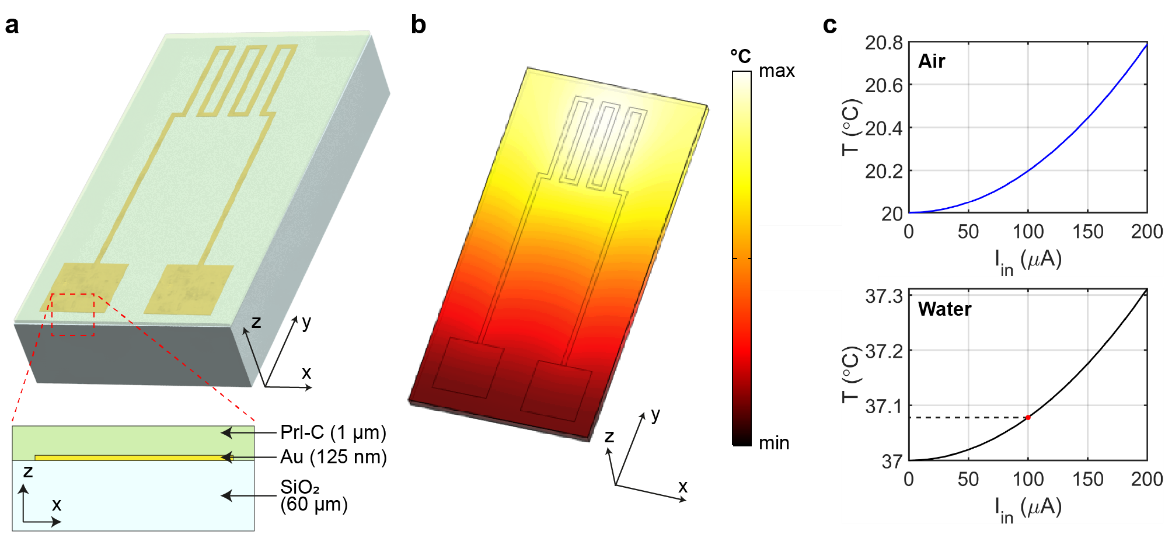
***

## **Calibration of the µRTD**

**Figure S3**. Residuals of the data plot in Figure 2c


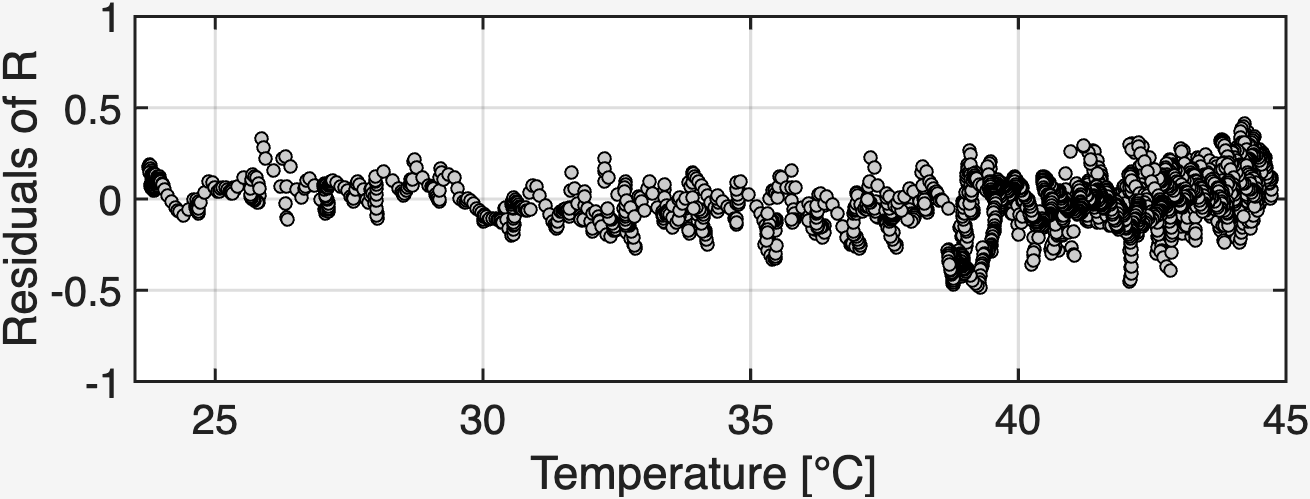


**Figure S4.** Extracted Resistance vs Temperature calibration curves for n = 3 distinct devices. Extended values report in Table S3.


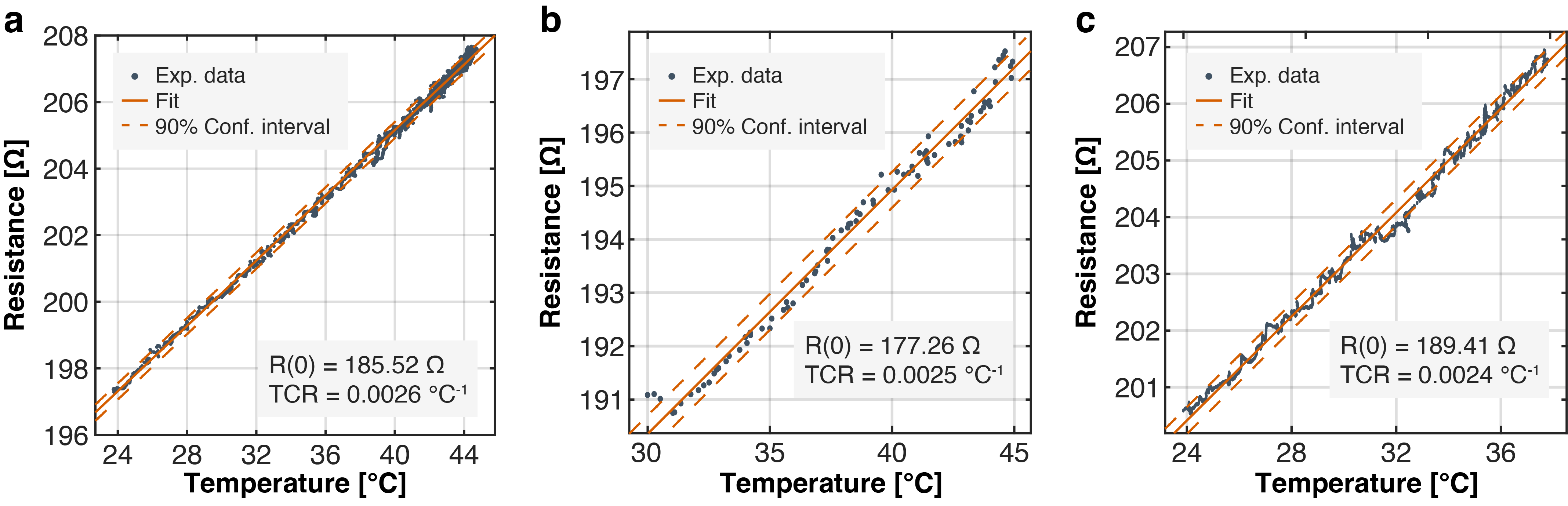


**Table S3. Summary of the key metrics extracted from the three μRTD - TF devices.** For R(0) and TCR, the best-fit value and the corresponding 90% confidence interval are listed. The last two rows show the mean and standard deviation across devices.

| Device # | *R*(0) [Ω] | Conf. int. [Ω] | *TCR* [°C^-1^] | Conf. int. [°C^-1^] | *S* [Ω/°C] | *A* [°C] |
| --- | --- | --- | --- | --- | --- | --- |
| #1 | 185.52 | [185.49 185.55] | 0.00265 | [0.00264 0.00265] | 0.491 | 0.21 |
| #2 | 177.26 | [176.88 177.65] | 0.00249 | [0.00243 0.00255] | 0.442 | 0.21 |
| #3 | 189.41 | [189.36 189.44] | 0.00243 | [0.00242 0.00244] | 0.460 | 0.33 |
| **Mean** | 184 | - | 0.00252 | - | 0.464 | 0.25 |
| **Std** | 6 | - | 0.00012 | - | 0.025 | 0.06 |

**Figure S5. Accuracy** as a function of **(a)** the applied Butterworth filter time constant τ_c_ and **(b)** the related cut-off frequency f_c_=1/τ_c_.


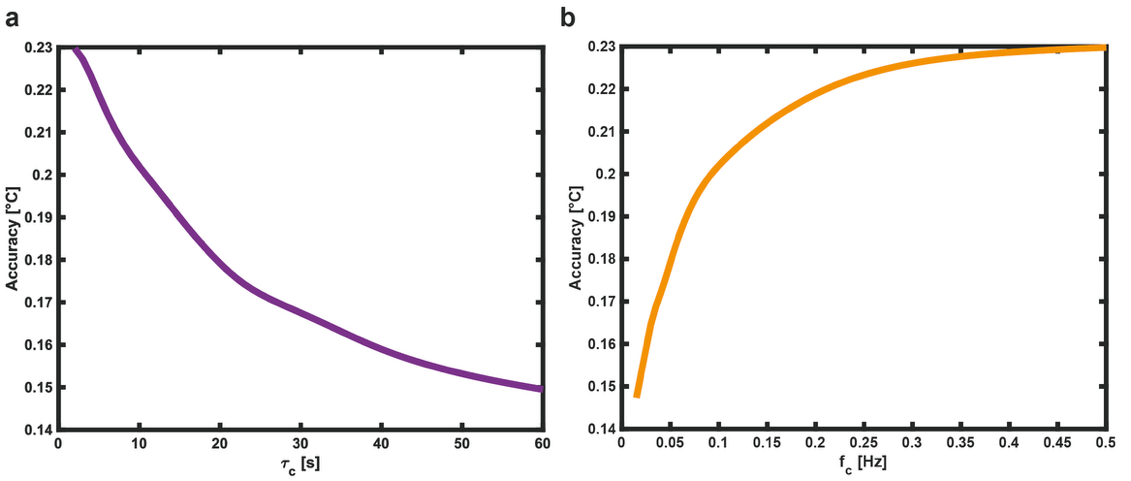


**Figure S6. Characterization of photoelectric artifacts** as a function of power density.


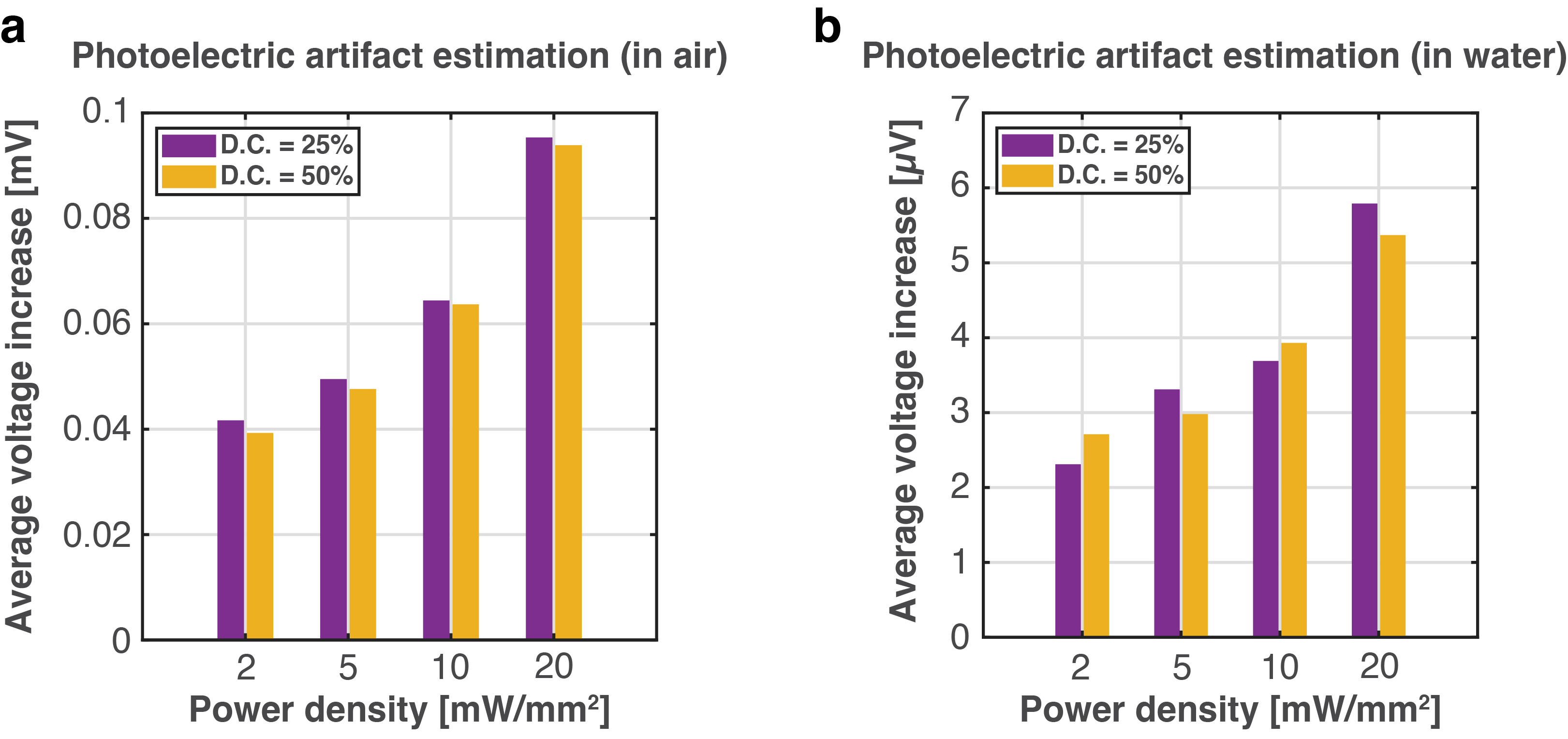


**Figure S7.** **Calibration with and without photoelectric artifacts. (a)** Resistance VS Temperature calibration curve of the raw data without filtering the photoelectric artifacts. **(b)**Table summarizing the characteristic parameters values before and after filtering, with the relative percentage difference.


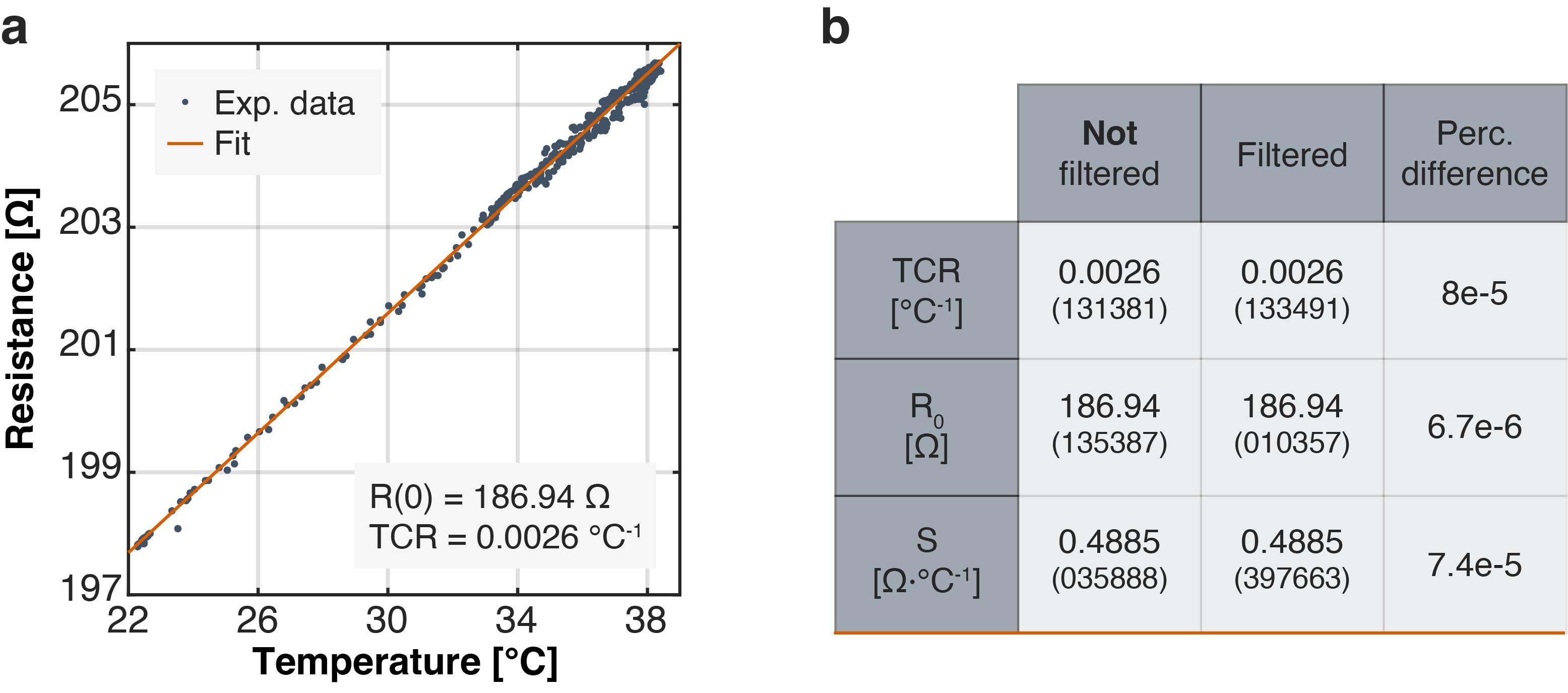


## **Raw data in vivo and Frequency-domain analysis**

**Figure S8. Raw data used to extract the variation of local brain temperature** while delivering light at different pulse durations and power densities displayed in Figure 3c-e. Colored lines represent the filtered signal and are reported for comparison.


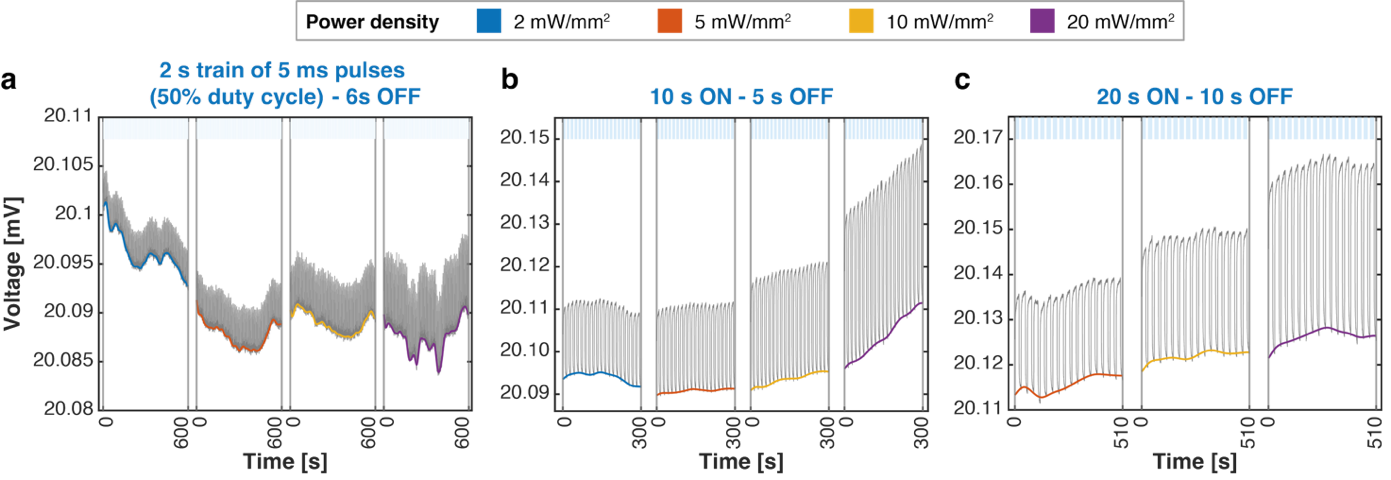


**Figure S9. Frequency-domain analysis of in vivo μRTD signals and effect of low-pass filtering. (a)**Amplitude spectra (FFT magnitude) of the raw (blue) and low-pass–filtered (red, τc = 30 s) μRTD signal recorded in vivo during the 20 mW/mm² illumination protocol corresponding to Fig. 4c. The inset shows a zoom of the 0–0.2 Hz range. **(b)**Power spectral density (PSD) of the same dataset for the raw (solid) and filtered (dashed) signals, together with the group delay of the digital low-pass filter (orange). The vertical dashed line marks 0.01 Hz. **(c-d)** Same analysis as in (a,b) for the μRTD signal acquired during the 20 mW/mm² protocol showing clear light-induced heating (Figure 4d).


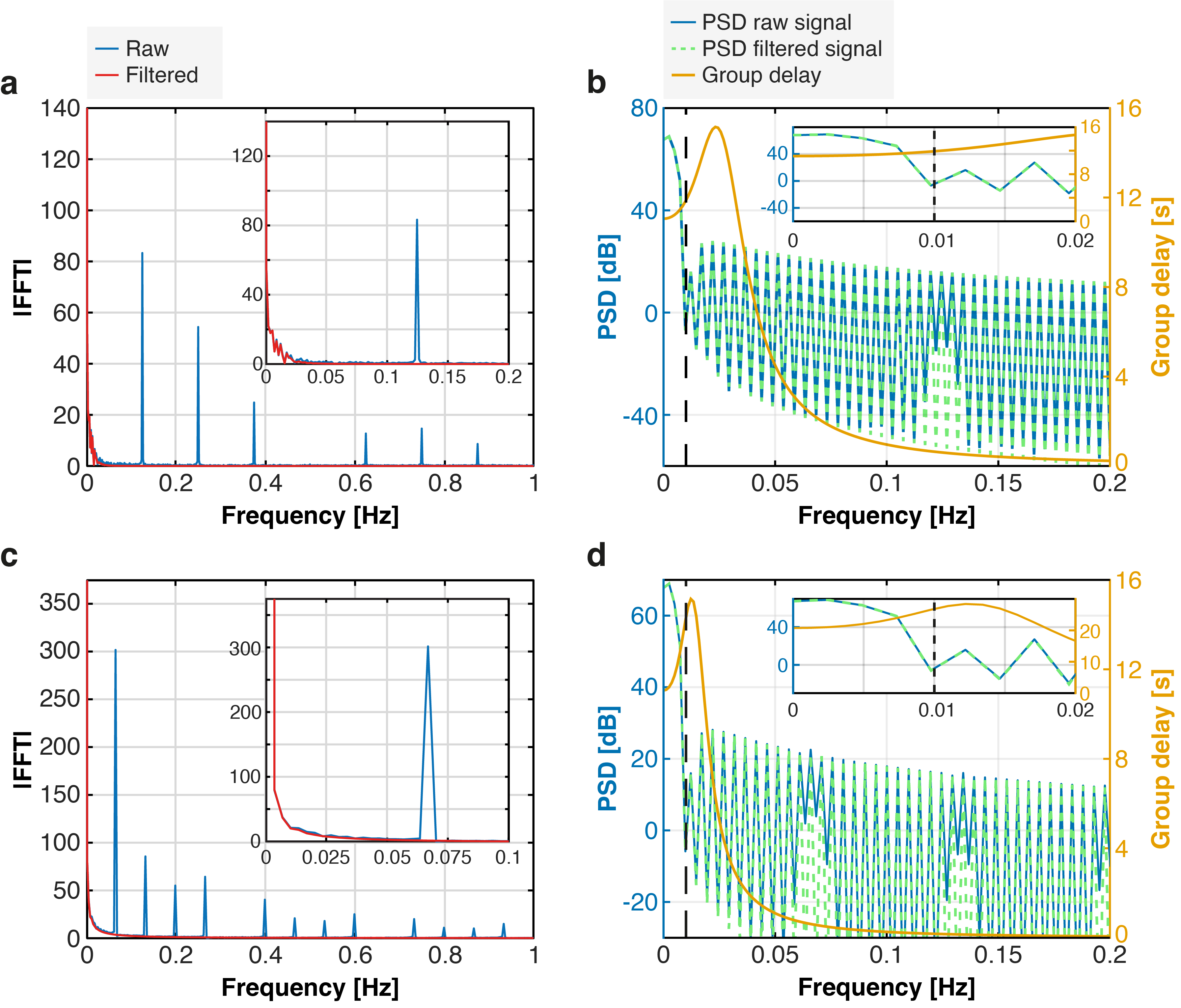

Supplement: Supplementary file 1 — Supporting File: adma72918‐sup‐0001‐SuppMat.docx [file ADMA-38-e19655-s001.docx]
